# Intraoperative endoluminal pyloromyotomy as a novel approach to reduce delayed gastric emptying after pylorus-preserving pancreaticoduodenectomy—a retrospective study

**DOI:** 10.1007/s00423-020-02008-5

**Published:** 2020-10-14

**Authors:** Matthias C. Schrempf, David R. M. Pinto, Johanna Gutschon, Christoph Schmid, Michael Hoffmann, Bernd Geissler, Sebastian Wolf, Florian Sommer, Matthias Anthuber

**Affiliations:** 1 Department of General, Visceral and Transplant Surgery, University Hospital Augsburg, Stenglinstrasse 2, Augsburg, 86156 Germany; 2 Department of Hematology and Oncology, University Hospital Augsburg, Stenglinstrasse 2, 86156 Augsburg, Germany

**Keywords:** Pylorus-preserving pancreaticoduodenectomy, Delayed gastric emptying, Pyloromyotomy, Pancreatic cancer

## Abstract

**Background:**

Delayed gastric emptying (DGE) is one of the most common complications after pylorus-preserving partial pancreaticoduodenectomy (ppPD). The aim of this retrospective study was to assess whether an intraoperative pyloromyotomy during ppPD prior to the creation of duodenojejunostomy reduces DGE.

**Methods:**

Patients who underwent pylorus-preserving pancreaticoduodenectomy between January 2015 and December 2017 were divided into two groups on the basis of whether an intraoperative pyloromyotomy was performed (pyloromyotomy (PM) group) or not (no pyloromyotomy (NP) group). The primary endpoint was DGE according to the ISGPS definition. The confirmatory analysis of the primary endpoint was performed with multivariate analysis.

**Results:**

One hundred and ten patients were included in the statistical analysis. Pyloromyotomy was performed in 44 of 110 (40%) cases. DGE of any grade was present in 62 patients (56.4%). The DGE rate was lower in the PM group (40.9%) compared with the NP group (66.7%), and pyloromyotomy was associated with a reduced risk for DGE in univariate (OR 0.35, 95% CI 0.16–0.76; *P* = 0.008) and multivariate analyses (OR 0.32, 95% CI 0.13–0.77; *P* = 0.011). The presence of an intra-abdominal complication was an independent risk factor for DGE in the multivariate analysis (OR 5.54, 95% CI 2.00–15.36; *P* = 0.001).

**Conclusion:**

Intraoperative endoluminal pyloromyotomy during ppPD was associated with a reduced risk for DGE in this retrospective study. Pyloromyotomy should be considered a simple technique that can potentially reduce DGE rates after ppPD.

## Introduction

Partial pancreaticoduodenectomy (PD) is the standard treatment for resectable tumors of the pancreatic head, the ampulla of Vater, and the distal common bile duct. In the 1970s, Traverso et al. [1, 2] introduced a pylorus-preserving modification (ppPD) which has been shown to be equally effective compared with the classical PD with regard to long-term survival and tumor recurrence. In-hospital mortality rates are less than 5% in high-volume centers, but perioperative morbidity remains high for PD and ppPD [3, 4]. One of the most common complications after either PD or ppPD is delayed gastric emptying (DGE) with an incidence of up to 61% [5]. Although DGE is not a lethal complication, it is associated with longer hospital stay, higher costs, and reduced quality of life [6, 7].

In 1985, Warshaw and Torchiana [8] first described DGE after PD. In 2007, the International Study Group of Pancreatic Surgery (ISGPS) proposed a definition and a grading system based on clinical parameters in order to standardize the term DGE which had been inconsistently defined by various authors [9]. The ISGPS definition represents the most widely accepted definition today.

While mild DGE usually resolves without further treatment, severe DGE may require noninvasive or invasive treatment. Commonly used treatments for DGE include gastric decompression via nasogastric tube, parenteral nutrition, use of prokinetics, and interventional treatment [10, 11]. The practices vary widely between institutions, and evidence is low.

The etiology of DGE remains largely unknown. Intra-abdominal complications such as anastomotic leakage, pancreatic fistula, and formation of hematoma or abscess are associated with a higher incidence of DGE in several studies [10, 12–14]. Therefore, the occurrence of DGE in the absence of intra-abdominal complications has been referred to as “primary DGE” by some authors in order to distinguish cases of DGE with and without accompanying intra-abdominal complications [5].

DGE has been attributed to spasm of the pyloric muscle, devascularization of the pylorus, and postoperative hormonal changes, although the underlying mechanisms remain poorly understood [15–19].

Numerous studies investigated modifications of the surgical procedure including the route of reconstruction, pyloric dilatation, and pyloric resection and their impact on DGE with conflicting results [5, 15, 20–24]. Larger randomized trials and meta-analyses using the ISGPS definition were unable to show an association between the investigated modification of the procedure and DGE [5, 20, 21, 25].

Under the assumption that pyloric spasm or dysregulation plays a role in the development of postoperative DGE, we added an intraoperative endoluminal pyloromyotomy during ppPD for some of our patients. In this retrospective study, we provide a description of the technique and present our initial experience.

## Methods

This study was conducted at the Department of General, Visceral and Transplant Surgery at University Hospital Augsburg, Germany, as a single-center retrospective study with a superiority hypothesis (intraoperative pyloromyotomy is associated with less DGE compared with no intraoperative pyloromyotomy). The study was approved by the Ethics Committee of Ludwig Maximilian University (LMU), Munich (reference number 17-620UE), and conducted in accordance with the Declaration of Helsinki.

### Study population and definitions

We identified all patients who underwent pylorus-preserving pancreaticoduodenectomy irrespective of the underlying diagnosis at our institution between January 2015 and December 2017 from the institutional electronic database. Electronic health records were reviewed, and perioperative data were extracted. Complications, comorbidities, operative data, and patient characteristics were collected from the database, including age at the time of surgery and sex. All operative reports were reviewed, and patients were divided into two groups according to the intraoperative handling of the pyloric muscle: those who underwent intraoperative pyloromyotomy (pyloromyotomy (PM) group) prior to the creation of the duodenojejunostomy and those who did not receive intraoperative pyloromyotomy (no pyloromyotomy (NP) group).

The ISGPS definition of DGE was applied for this study [9]. DGE grade A was present if the nasogastric tube (NGT) was still in place or reinserted between postoperative days (POD) 4 and 7 or if the patient was unable to tolerate a solid oral diet by POD 7. Patients suffered from grade B DGE if the NGT was still in place or reinserted between POD 8 and 14 or if patients were unable to tolerate a solid oral diet by POD 14. If the NGT was still in place or reinserted after POD 14 or if patients were unable to tolerate a solid diet by POD 21, DGE was assessed as grade C in accordance with the ISGPS definition. The Clavien-Dindo classification [26] was used for grading of complications. The presence of a postoperative pancreatic fistula (POPF) was diagnosed by lipase measurements from the fluid output via intra-abdominal drains, or if lipase measurements were not performed, a clinical diagnosis was made by the surgeon in charge based on the appearance of drain fluids. The grading of the POPF (biochemical leak, grade B or C) was done in accordance with the 2016 update of the ISPGS definition of POPF [27].

### Description of surgical technique

**Fig. 1:**
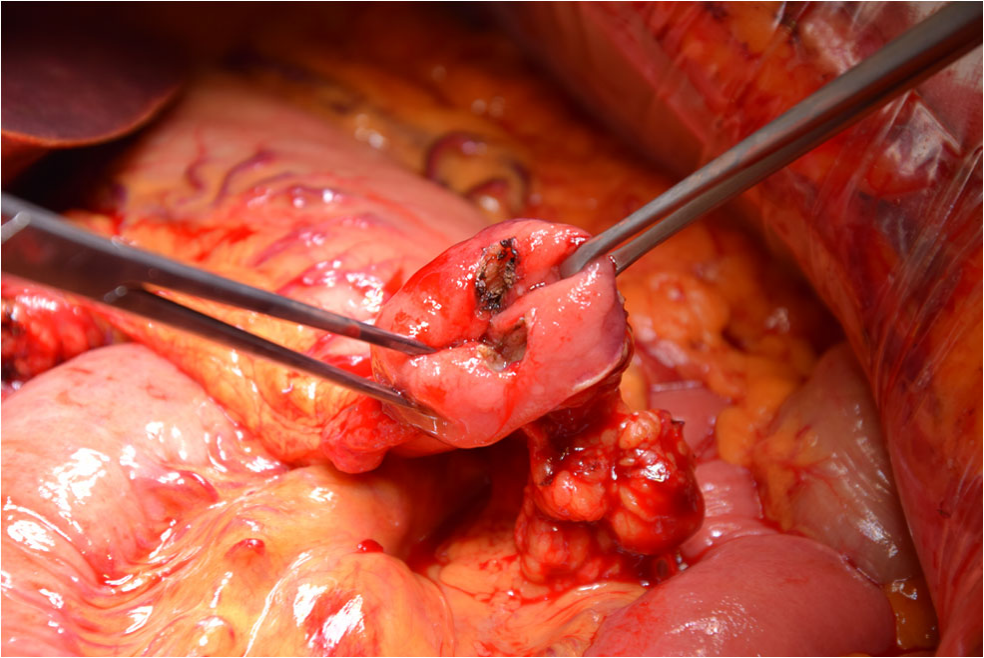
Intraoperative endoluminal pyloromyotomy

The standard surgical procedure was a pylorus-preserving PD. The duodenum was divided into 2 to 4 cm distal to the pylorus. Pancreaticojejunostomy and hepaticojejunostomy were performed using end-to-side anastomoses. Prior to the creation of the duodenojejunostomy, one of two different surgical maneuvers was routinely performed at the discretion of the surgeon in charge. An intraoperative endoluminal pyloromyotomy was performed using electrocautery to transect the mucosa, the submucosa, and the circular pyloric muscle anteriorly and posteriorly at the 12 and 6 o’clock positions (Fig. 1), or if pyloromyotomy was not performed, a Gross-Maier dressing forceps was used to apply atraumatic multidimensional stretching to the pyloric muscle prior to the creation of duodenojejunostomy. In all patients, reconstruction was performed with an omega loop in an antecolic fashion and a side-to-side Braun jejunojejunostomy approximately 15 cm distal to the duodenojejunostomy. Based on the internal standard, subcutaneous octreotide at a dose of 100 μg was administered intraoperatively at the time of the creation of the duodenojejunostomy and octreotide injections were continued three times daily until postoperative day 4.

### Endpoints

The primary endpoint was the rate of DGE as defined by the ISGPS in the PM and NP groups.

Secondary endpoints were the DGE grade, length of hospital stay, in-hospital mortality, and rate of postoperative complications.

### Statistical analysis

Continuous data is presented as mean ± standard deviation or median with interquartile range, depending on the distribution. Categorical data is presented as numbers with percentages. Approximately normally distributed continuous variables were compared using the independent *t* test. Nonnormally distributed continuous variables were compared using the Mann-Whitney *U* test. Categorical data was compared using the *χ*^2^ test. Fisher’s exact test was used for categorical data when the requirements for the *χ*^2^ test were not met. A two-sided *P* < 0.05 was considered significant. Baseline characteristics (age, BMI, sex, chronic renal insufficiency, diabetes mellitus, previous abdominal surgery, malignant disease, ASA III or higher) and early postoperative intra-abdominal complications (chyle leak, intra-abdominal fluid collection, pancreatic fistula, and the presence of at least one complication arising from an intra-abdominal focus) were tested for potential association with DGE in univariate analysis. Risk factors with a potential association (*P* < 0.15) with DGE in univariate analysis and risk factors which had shown an association with DGE in multivariate analysis of prospective studies (BMI, POPF, sex, benign pathology, intra-abdominal complication) [10, 13, 20, 28] were included in multivariate analysis. The confirmatory analysis of the primary endpoint was performed with multivariate analysis (binary logistic regression) including all risk factors with potential association with DGE (*P* < 0.15) and risk factors which had shown an association with DGE in multivariate analysis of prospective studies. Statistical analyses were undertaken using SPSS® for Windows®, version 24 (IBM, Armonk, NY, USA).

## Results

Between January 2015 and December 2017, a total of 114 patients underwent ppPD without vascular reconstruction at our institution. Four patients were excluded from the analysis. One patient was excluded because he had undergone choledochojejunostomy previous to ppPD, and three patients were excluded because of incomplete documentation. All operations were performed by a total of nine surgeons.

Of 110 patients that were included in the statistical analysis, 40% (44 of 110 patients) received pyloromyotomy and 60% (66 of 110 patients) did not receive pyloromyotomy prior to the creation of the duodenojejunostomy. Baseline demographics and clinical characteristics were similar between the two groups (Table 1).

**Table 1:** Demographics and clinical characteristics

Characteristic	PM (*n* = 44)	NP (*n* = 66)	*P*
Sex, *n* (%)			
Female	22 (50.0%)	29 (43.9%)	0.532
Male	22 (50.0%)	37 (56.1%)	
BMI ≥ 30 kg/m^2^, *n* (%)	3 (6.8%)	9 (13.6%)	0.356
Chronic renal insufficiency, *n* (%)	7 (15.9%)	8 (12.1%)	0.571
Diabetes mellitus, *n* (%)	8 (18.2%)	13 (19.7%)	0.843
Previous abdominal surgery, *n* (%)	15 (34.1%)	21 (31.8%)	0.803
ASA, *n* (%)			
I	2 (4.5%)	4 (6.1%)	0.621
II	22 (50.0%)	28 (42.4%)	
III	20 (45.5%)	34 (51.5%)	
Histology, *n* (%)			
Malignant	37 (84.1%)	58 (87.9%)	0.571
Benign	7 (15.9%)	8 (12.1%)	
Age	69.6 ± 10.8	68.8 ± 11.6	0.802

Data are mean ± SD or *n* (%)

*PM* pyloromyotomy, *NP* no pyloromyotomy, *BMI* body mass index, *ASA* American Society of Anesthesiologists

### Delayed gastric emptying

In the study population, DGE of any grade was present in 62 patients (56.4%). The rate of DGE was 40.9% in the PM group (18 of 44 patients) and 66.7% in the NP group (44 of 66 patients). Patients who underwent pyloromyotomy had a lower risk for DGE in univariate analysis (OR 0.315, 95% CI 0.16–0.76; *P* = 0.008). Grade A DGE was the most common type of DGE followed by grades B and C. The distribution of DGE grades did not differ significantly between the PM and NP groups (*P* = 0.465). Grades A and C were more common in the NP group (PM 22.7% vs. NP 39.4% and PM 4.5% vs. NP 13.6%, respectively) whereas grade B was equally common in both groups (13.6%). The intraoperatively placed NGT was required for a shorter duration (*P* = 0.019) in the PM group (mean 1.49 days) compared with the NP group (mean 4.11 days). In the PM group, 7 patients (15.9%) required reinsertion of the NGT whereas 15 patients (22.7%) required reinsertion in the NP group (*P* = 0.381). The outcomes are shown in Table 2.

**Table 2:** Outcomes

Characteristic	PM (*n* = 44)	NP (*n* = 66)	*P*
DGE, *n* (%)	18 (40.9%)	44 (66.7%)	0.008
DGE grade*, *n* (%)			
A	10 (22.7%)	26 (39.4%)	0.465
B	6 (13.6%)	9 (13.6%)	
C	2 (4.5%)	9 (13.6%)	
DGE grades B and C only, *n* (%)	8 (18.2%)	18 (27.3%)	0.272
Operating time, median (IQR) (min)	258 (220–290)	312 (268–353)	< 0.001
Estimated blood loss, median (IQR) (mL)	450 (300–675)	600 (400–1000)	0.150
Removal of the first NGT (days)	1.49 ± 1.6	4.11 ± 9.5	0.019
Solid food intake (days)	9.4 ± 5.9	12.6 ± 12.8	0.108
Reinsertion of NGT, *n* (%)	7 (15.9%)	15 (22.7%)	0.381
Reoperation, *n* (%)	5 (11.4%)	10 (15.2%)	0.571
Postoperative stay (days)	20.4 ± 9.9	24.1 ± 14.5	0.150

Data are mean ± SD or *n* (%) or median (IQR)

*PM* pyloromyotomy, *NP* no pyloromyotomy, *DGE* delayed gastric emptying, *NGT* nasogastric tube, *IQR* interquartile range, *SD* standard deviation

*Only patients with DGE are included in the analysis

### Secondary outcomes and complications

Operating time was significantly lower in the PM group (PM: median 258 min vs. NP: median 312 min; *P* < 0.001). Reoperation rates were similar between both groups (PM, 6 of 44; 13.6% vs. NP, 10 of 66; 15.2%; *P* = 0.825). There was no difference in intraoperative blood loss (*P* = 0.150) and length of hospital stay (*P* = 0.150) between both groups.

Postoperative complications are shown in Table 3. The overall in-hospital mortality rate in the study population was 2.7% (3 of 110 patients) with no difference between both groups (*P* = 0.273). Relaparotomy was performed in 15 of 110 patients (five cases of postoperative hemorrhage, four cases of pancreatic fistula grade C, two cases of bile leak, two cases of wound dehiscence, and one case of exploratory laparotomy). In the overall population, 38 of 110 patients (34.5%) developed an intra-abdominal complication. Intra-abdominal complication rates and major complications (Clavien-Dindo III or higher) did not differ between the PM and NP groups (*P* = 0.368 and *P* = 0.190). All other complications did not differ between both groups (Table 3).

**Table 3:** Postoperative complications

Characteristic	PM (*n* = 44)	NP (*n* = 66)	*P*
In-hospital mortality, *n* (%)	0 (0%)	3 (4.5%)	0.273
Intra-abdominal complication, *n* (%)	13 (29.5%)	25 (37.9%)	0.368
Complication Clavien-Dindo III or higher, *n* (%)	9 (20.5%)	21 (31.8%)	0.190
Biochemical leak, *n* (%)	0	3 (4.5%)	0.273
POPF, *n* (%)	6 (13.6%)	8 (12.1%)	0.815
Grade B, *n* (%)	4 (9.1%)	5 (7.6%)	1.00
Grade C, *n* (%)	2 (4.5%)	3 (4.5%)	1.00
Intra-abdominal fluid collection, *n* (%)	2 (4.5%)	3 (4.5%)	1.00
Bile leak, *n* (%)	3 (6.8%)	5 (7.6%)	1.00
Chyle leak, *n* (%)	2 (4.5%)	1 (1.5%)	0.563
Postoperative hemorrhage, *n* (%)	5 (11.4%)	8 (12.1%)	0.904
Leakage of duodenojejunostomy, *n* (%)	0 (0%)	1 (1.5%)	1.00
Pulmonary aspiration and pneumonia, *n* (%)	0 (0%)	4 (6.1%)	0.148
Pulmonary embolism, *n* (%)	0 (0%)	1 (1.5%)	1.00
Surgical site infection, *n* (%)	3 (6.8%)	7 (10.6%)	0.737
Wound dehiscence, *n* (%)	1 (2.3%)	2 (3.0%)	1.00
Portal vein thrombosis, *n* (%)	1 (2.3%)	2 (3.0%)	1.00
Urinary tract infection, *n* (%)	0 (0%)	3 (4.5%)	0.273
Liver failure, *n* (%)	0 (0%)	1 (1.5%)	1.00
NSTEMI, *n* (%)	0 (0%)	1 (1.5%)	1.00

Data are *n* (%)

*PM* pyloromyotomy, *NP* no pyloromyotomy, *POPF* postoperative pancreatic fistula, *NSTEMI* non-ST elevation myocardial infarction

### Risk factors for DGE and multivariate analysis

Demographic factors and early postoperative complications with potential association with DGE (*P* < 0.15 in univariate analysis) were tested for association with DGE (Table 4). The presence of an intra-abdominal complication showed a strong association with the occurrence of DGE in univariate analysis (OR 7.38, 95% CI 2.58–21.16; *P* < 0.001) and remained an independent risk factor for DGE even after correcting for the intraoperative handling of the pyloric muscle (OR 5.54, 95% CI 2.00–15.36; *P* = 0.001). Pyloromyotomy was associated with a risk reduction for DGE in the multivariate analysis (OR 0.32, 95% CI 0.13–0.77; *P* = 0.011).

**Table 4:** Risk factors and protective factors for DGE

Variable	DGE (*n* = 62; 56.4%)	No DGE (*n* = 48; 43.6%)	Univariable *P*	Multivariable OR (95% CI)	Multivariable *P*
Age	69.8 (10.8)	68.2 (11.1)	0.323	–	
Diabetes mellitus, *n* (%)	11 (17.7%)	10 (20.8%)	0.682	–	
Female*, *n* (%)	31 (50%)	20 (41.7%)	0.385	1.86 (0.76–4.55)	0.175
BMI ≥ 30 kg/m^2^*, *n* (%)	9 (14.5%)	3 (6.3%)	0.168	1.71 (0.38–7.69)	0.485
ASA III or higher, *n* (%)	29 (46.8%)	25 (52.1%)	0.581	–	
Chronic renal insufficiency, *n* (%)	11 (17.7%)	4 (8.3%)	0.154	–	
Previous abdominal surgery, *n* (%)	21 (33.9%)	15 (31.3%)	0.771	–	
Malignant disease*, *n* (%)	54 (87.1%)	41 (85.4%)	0.799	1.21 (0.35–4.24)	0.767
Chyle leak, *n* (%)	2 (3.2%)	1 (2.1%)	1.00	–	
Intra-abdominal fluid collection, *n* (%)	2 (3.2%)	3 (6.3%)	0.651	–	
POPF, *n* (%)	11 (17.7%)	3 (6.3%)	0.073	2.60 (0.52–13.04)	0.246
Intra-abdominal complication, *n* (%)	31 (50.0%)	7 (14.6%)	< 0.001	5.54 (2.00–15.36)	*0.001*
Pyloromyotomy performed, *n* (%)	18 (29.0%)	26 (54.2%)	0.008	0.32 (0.13–0.77)	*0.011*

Data are mean ± SD or *n* (%)

*DGE* delayed gastric emptying, *BMI* body mass index, *ASA* American Society of Anesthesiologists, *POPF* postoperative pancreatic fistula

*Additionally included in multivariate analysis because of association with DGE in multivariate analysis of prospective studies [10, 13, 20, 28]

Since we noticed a significant difference in operating time between the PM and NP groups, we performed an additional multivariate analysis with the inclusion of the potential risk factor operating time. Despite the inclusion of operating time in the multivariate analysis, pyloromyotomy remained associated with a reduced risk for DGE (OR 0.36, 95% CI 0.14–0.92; *P* = 0.032). Operating time itself was not associated with DGE in multivariate analysis (*P* = 0.36).

In order to assess the rate of DGE in the absence of an intra-abdominal complication, which is also referred to as primary DGE, we performed a subgroup analysis of the 72 patients without an intra-abdominal complication. The overall rate of DGE was 43.1% (31 of 72 patients) within the subgroup. In the PM group, 29.0% of patients (9 of 31 patients) compared with 53.7% (22 of 41 patients) in the NP group developed a DGE (OR 0.35, 95% CI 0.13–0.95; *P* = 0.037).

## Discussion

This retrospective analysis showed a significant difference in DGE rates between patients in whom an intraoperative pyloromyotomy was performed and patients who did not receive an intraoperative pyloromyotomy. We did not notice any complications in this study resulting directly from the incision of the pyloric muscle such as bleeding from the pyloric muscle. The overall rate of DGE in this series was 56.9% and on the higher end of the reported range of DGE rates but is in line with DGE rates from prospective trials [5, 6, 16] underlining that DGE is a frequent and burdening complication after PD. Although we did not apply a standardized protocol for postoperative return to an oral diet at the time of the study, it was common practice to remove the NGT if no or limited reflux was present and return to a liquid diet followed by a solid diet as soon as clinically feasible.

Intra-abdominal complications were strongly associated with DGE and remained an independent risk factor even after correction for the surgical approach. Postoperative intra-abdominal complications have been previously discussed as a possible causal factor in the development of DGE [20, 29]. Some authors even suggested to distinguish between “primary DGE” in the absence of intra-abdominal complications and DGE accompanied by complications [5]. Therefore, we performed a subgroup analysis in order to get a better understanding of the effect of our surgical modification. Among patients without any intra-abdominal complications, we also found a reduced risk for DGE in patients who underwent pyloromyotomy (OR 0.35, 95% CI 0.13–0.95; *P* = 0.037).

The concept of pyloromyotomy for the reduction of DGE in ppPD has been studied previously. Kim et al. [16] performed a Fredet-Ramstedt-type pyloromyotomy in combination with an antroplasty [30] in a series of 47 consecutive ppPD patients. They reported a DGE incidence of 2.2% with DGE defined as an inability to tolerate any oral intake including a liquid diet for three consecutive days in the absence of any attributable complications. Although the low incidence in the series by Kim et al. can be partially explained with the strict individual definition of DGE, a comparison to patients from the same institution treated before the introduction of the Fredet-Ramstedt pyloromyotomy by the same authors showed a reduction in DGE rates with pyloromyotomy. Additionally, the successful treatment of gastroparesis with underlying increased pyloric tone and pylorospasm with peroral endoscopic endoluminal pyloromyotomy [31–33] supports the idea to apply an intraoperative pyloromyotomy for the reduction of DGE.

Two Japanese trials and one German randomized controlled trial investigated the association between pyloric resection and DGE. Kawai et al. [34] found a significant reduction in DGE incidence between pylorus-resecting and pylorus-preserving pancreaticoduodenectomy (4.5% vs. 17.2%; *P* = 0.02). Matsumoto et al. [35] excluded patients with pancreatic cancer from the trial and did not find an association between pylorus resection and incidence of DGE. The PROPP trial by Hackert et al. [20] which represents the most recent and largest RCT investigating the effect of pylorus resection did not find a difference in DGE incidence or severity between the two groups. The results from the PROPP trial argue against pyloric dysfunction as the main mechanism in the development of DGE and favor a multifactorial etiology. Arguably, the results from the PROPP trial partially question the concept of pyloromyotomy for the reduction of DGE which is largely based on the idea of a postoperative impaired pyloric function. These conflicting results underline our incomplete understanding of the etiology of DGE, and further research is required to fully elucidate the mechanisms causing postoperative DGE.

As with most retrospective studies, this study has several limitations. These include incomplete documentation, interpretation bias, and variability in the clinical management of patients in the postoperative period. The fact that pyloromyotomy was performed at the discretion of the surgeon in charge might have introduced a performance bias since the participating surgeons had different levels of training. Approximately one-third of participating surgeons mostly performed pyloromyotomy while two-thirds preferred to apply stretching to the pyloric muscle. A difference in operating times between the PM and NP groups might be an indication of a higher level of training in the PM group, but operating time was not associated with DGE in the multivariate analysis.

## Conclusion

Despite limitations, this study delivered positive results for a simple technique that could potentially lower the incidence of DGE after PD. These results encouraged us to further investigate our findings and the impact of intraoperative pyloromyotomy on DGE and quality of life in a randomized prospective study which has been registered at the German Registry of Clinical Trials (DRKS Nr. 00013503) and is currently enrolling patients.
